# Diagnosis and management of nonallergic rhinitis with eosinophilia syndrome using cystatin SN together with symptoms

**DOI:** 10.1016/j.waojou.2020.100134

**Published:** 2020-06-17

**Authors:** Yifan Meng, Bing Yan, Yang Wang, Di Wu, Luo Zhang, Chengshuo Wang

**Affiliations:** a Department of Otolaryngology Head and Neck Surgery, Beijing TongRen Hospital, Capital Medical University, Beijing, 100730, China; b Beijing Key Laboratory of Nasal Diseases, Beijing Institute of Otolaryngology, Beijing, 100005, China; c Department of Allergy, Beijing TongRen Hospital, Capital Medical University, Beijing, 100730, China

**Keywords:** Cystatin SN, Diagnosis, Nonallergic rhinitis with eosinophilia syndrome (NARES), Treatment efficacy, NARES, nonallergic rhinitis with eosinophilia syndrome, CR, chronic rhinitis, AR, allergic rhinitis, NAR, nonallergic rhinitis, IgE, immunoglobulin E, LAR, local allergic rhinitis, IR, idiopathic rhinitis, GINA, Global Initiative for Asthma guidelines, VAS, visual analogue scale, LE, local eosinophils, SIgE, specific IgE, ROC, receiver operating characteristic, AUC, area under curve, VIF, variance inflation factor, ECRSwNP, eosinophilic chronic rhinosinusitis with nasal polyps, INS, intranasal corticosteroid, GICA, gold immunochromatographic assay

## Abstract

**Background:**

The diagnosis and treatment of nonallergic rhinitis with eosinophilia syndrome (NARES) remain controversial. The aim of this study was to evaluate whether Cystatin SN together with symptoms can be used to diagnose NARES and to measure the efficiency of medical treatment.

**Methods:**

Seventy-five patients with chronic rhinitis (CR) and 18 control subjects were enrolled. Their clinical characteristics were reviewed and laboratory parameters were evaluated. The concentration of Cystatin SN in nasal secretions was determined using the enzyme-linked immunosorbent assay. The histological assessment of Cystatin SN in the nasal mucosa was conducted by hematoxylin and eosin staining. The logistic regression and receiver operating characteristic curves were used to assess the predictive value of parameters for NARES.

**Results:**

Nasal obstruction, sneezing, loss of smell, and total visual analogue scale (VAS) score were significantly different among the patients with CR. In particular, olfaction score was higher in patients with NARES than in those without NARES (AR, LAR, or IR). Similarly, the Cystatin SN level was significantly different between the control subjects and patients with CR. After treatment for 2 weeks, the Cystatin SN level and VAS score were significantly decreased in the NARES group. The accuracy of Cystatin SN together with local sIgE and loss of smell to diagnose NARES was up to 0.987 (sensitivity, 100%; specificity, 93.1%).

**Conclusions:**

Cystatin SN with local sIgE and loss of smell may serve as one of the reliable and alternative biomarkers for the diagnosis of NARES and be used to evaluate disease severity and NARES treatment efficacy.

## Introduction

Chronic rhinitis (CR) is defined as a symptomatic inflammation of the nasal mucosa, with morbidity as high as 30% of the total population.^1^^,^^2^ CR is generally subcategorized into the following 2 phenotypes: allergic rhinitis (AR) and nonallergic rhinitis (NAR).^3^^,^^4^ AR is a very common disorder worldwide and has been thoroughly studied.^5^ However, NAR is a disease that is usually underestimated although approximately 200 million people are affected by this disease worldwide.^2^ Nonallergic rhinitis with eosinophilia syndrome (NARES), one of the most important phenotypes of NAR, affects around 2%–33% patients with CR worldwide.^6^, ^7^, ^8^

In 1981, Jacob et al.,^9^ first described NARES as a condition with symptoms of anosmia, sneezing, watery rhinorrhea, and nasal pruritus, but presenting negative results in the allergy test. Neither elevated total immunoglobulin E (IgE) nor specific IgE is observed in the nasal secretions of patients with NARES.^9^^,^^10^ NARES is a disease that is highly associated with the entire airway. A study suggested that it could be a precursor of nasal polyps, asthma, and aspirin-exacerbated respiratory disease.^11^ Although the pathophysiology of NARES is yet to be fully understood, chronic, nonspecific liberation of histamine and chronic eosinophilic nasal inflammation have been suggested as the 2 common pathogenic factors of the disease.^10^ By evaluating 20 patients with NARES, Moneret-Vautrin et al.,^12^ revealed a three-stage development process of the disease: (1) migration of eosinophils from the vessels into nasal secretions; (2) retention of eosinophils in the mucosae, and this might be linked to activation by an unknown origin; and (3) development of nasal micropolyposis and polyposis. This process indicates that eosinophils are pivotal in the pathophysiology of NARES. Therefore, most researchers diagnose NARES by the level of eosinophils in the nasal smear; however, although a high level of eosinophilic cells is a common feature, the diagnostic criteria of eosinophils vary from 5% to 25%.^7^^,^^8^^,^^13^, ^14^, ^15^ Hence, it is rather difficult to select diagnostic criteria in this wide range for eosinophils in the nasal smear. Furthermore, such a wide range does not aid in the assessment of medical efficacy, warranting a relatively stable biomarker to diagnose and treat NARES.

Cystatin, which can be classified as type 1 to type 4, is a member of the cysteine protease inhibitor superfamily. Type 2 cystatins include cystatins S, SA, and SN. Human cystatins S, SA, and SN are non-glycosylated proteins found in tears, urine, saliva, liver, seminal plasma, and muscle.^16^ Cystatin SN, which is encoded by *CST1* and found only in primates, is secreted into body fluids, such as saliva and tears in humans^17^^,^^18^ and is highly associated with the nasal disease. The *CST1* mRNA has been reported to be upregulated in the nasal epithelia of patients with Japanese cedar-specific and other seasonal allergic rhinitis during the pollen season.^19^^,^^20^ However, the role of Cystatin SN in NARES has not been investigated. Herein, we determined the level of Cystatin SN in the nasal secretion of patients with NARES and evaluated its capacity in diagnosing NARES and assessing medical treatment efficacy along with clinical characteristics.

## Methods

### Study design and subjects

This was a retrospective single-center study. Eighteen control subjects and 75 patients who were suspected to have rhinitis based on the presence of common symptoms such as nasal obstruction, rhinorrhea, sneezing, and itching were recruited from the allergy-rhinology out-patient clinic of our hospital. Each subject completed a questionnaire at recruitment, which was used to retrieve demographic data, nasal symptom severity, and asthma history. Allergic rhinitis (AR), local allergic rhinitis (LAR), idiopathic rhinitis (IR), and NARES were diagnosed according to the 2008 Allergic Rhinitis and its Impact on Asthma (ARIA) criteria.^1^ Asthma was diagnosed according to the Global Initiative for Asthma guidelines (GINA) 2014.^21^ Healthy subjects without any nasal disease were recruited as controls.

The exclusion criteria for the study included chronic rhinosinusitis and/or nasal polyposis as defined by the European position paper on rhinosinusitis and nasal polyps,^22^ any respiratory infection in the last 4 weeks, and a computed tomography scan showing opacification in the nasal cavity or sinuses. Patients who had taken systemic corticosteroids during the last 3 months, intranasal corticosteroids in the last 4 weeks, antihistamines in the last 2 weeks, and vasoconstrictors in the last 1 week were also excluded.

A combination of two sprays, 64 μg budesonide (Rhinocort; AstraZeneca AB, Cambridge, UK) in the morning (1 spray per nostril, total = 128 μg per day) and one tablet of 10 mg montelukast (Merck Sharp & Dohme Australia Pty., Ltd.) in the evening, was administered to the NARES group for 2 weeks. At the start and end of the treatment, the severity of nasal symptoms (including nasal congestion, rhinorrhea, itching, and sneezing) was assessed using a visual analogue scale (VAS).

The study was conducted in compliance with the Declaration of Helsinki and approved by the Medical Ethics Committee of our Hospital. All patients provided written informed consent before enrollment and data collection.

### Visual analogue scale

The severity of nasal symptoms, including nasal obstruction, anterior or posterior rhinorrhea (watery, mucous, or purulent), sneezing, and nasal itching, was recorded using a VAS score of 10 cm. Each symptom was categorized as “mild” (VAS: 0–3 cm), “moderate” (VAS: >3–7 cm), or “severe” (VAS: >7 cm).

### Evaluation of local eosinophils

Eosinophils in the nasal secretion (local eosinophils, LEs) were counted as following the method described by Webb et al.^23^ The relative number of eosinophils in the nasal smears was evaluated using the following five-point scale: 0 = none; 1 = few, scattered; 2 = moderate number; 3 = large clumps, not covering the field; and 4 = clumps covering the entire field. Patients with NAR with the score higher than 0 were classified as NARES.

### Histological evaluation of cystatin SN in the nasal mucosa

Nasal mucosa samples from each patient were processed for histological evaluation using standardized procedures. Paraffin-embedded samples were sectioned to 4-μm thick slices and stained with rabbit anti-human CST1 mAb (Abcam, Cambridge, UK). All stained samples were observed using a bright-field light microscope at 400 × magnification. A semiquantitative analysis of stained Cystatin SN was performed as described previously.^24^ For each section, 3 non-overlapping regions were scored and 5 sections in each group from different subjects were included.

### Collection of nasal cavity secretions

The nasal cavity secretions were collected bilaterally from each subject according to the method described previously.^25^ Briefly, nasal secretions were obtained by inserting a postoperative sinus sponge pack into each nostril for 5 min. The secretion was extracted from the sponge by adding 1 mL of 0.9% sodium chloride solution. All sponges were stored at 4 °C for 2–24 h, and then transferred to a 5-mL BD syringe (Becton, Dickinson and Company, Franklin Lakes, NJ) and centrifuged at 1500×*g* for 10 min at 4 °C. The supernatants were separated and stored in aliquots at −80 °C until further use.

### Serum sIgE and local sIgE measurements

Serum and local sIgE levels to common aeroallergens were determined using the fluoroenzyme immunosorbent assay (UniCAP, Uppsala, Sweden); with a value for serum sIgE ≥0.35 kUA/L regarded as positive. The sIgE examination was performed with a panel of allergens including tree pollen (willows, poplars, and elms), ragweed, mugwort, house dust mites (containing *Dermatophagoides pteronyssinus* and *D. farinae*), house dust, pet allergens (cats and dogs), cockroaches, mold allergens (*Penicillium notatum*, *Cladosporium, Alternaria*, *Aspergillus*, and *Candida albicans*), and *Humulus scandens*.

### Measurement of cystatin SN in the nasal secretion

Cystatin SN in nasal secretion was measured using a commercially available enzyme-linked immunosorbent assay (ELISA) kit (Cloud-Clone Corp, Wuhan, China). Before the assay, all samples were diluted 50-fold with 0.9% normal saline; the assay was conducted strictly according to the manufacturer's instructions. The concentration of Cystatin SN was normalized using the concentration of total protein, which was measured using the Enhanced BCA Protein Assay Kit from Beyotime (Nanjing, China).

### Statistical analysis

The clinical parameters between the groups were compared using Chi-square test. The concentration of Cystatin SN was analyzed using Kruskal-Wallis H test to assess significant intergroup variability among more than two groups. A paired *t*-test was used to compare data pre- and post-medical treatment. Correlation between two variables were determined using Spearman's analysis, where an *r*-value of >0.7, 0.5–0.7, and <0.5 indicated a high correlation, moderate correlation, and low correlation, respectively. Logistic regression was used to identify potential predictors of NARES. Hosmer-Lemeshow test was performed to assess the suitability of the models. The receiver operating characteristic (ROC) curve was used to calculate the sensitivity and specificity of the predictor. Data are expressed as median and interquartile range unless otherwise specified. Differences were considered significant if the *p*-value was <0.05. The AUCs were compared using MedCalc statistics software package (version 15.2, Ostend, Belgium). Other statistical analyses were performed using GraphPad Prism 8.0 software (GraphPad Software, Inc., La Jolla, CA) and SPSS for Windows version 22.0 (IBM Corp., Armonk, NY, USA).

## Results

### Differences in clinical characteristics and cystatin SN expression

There was no significant difference with regard to age and sex between the groups, and morbidity of asthma, rhinorrhea score, and nasal itching score among patients with CR. However, nasal obstruction score, sneezing score, olfaction score, and total VAS score were significantly different (Table 1). In particular, olfaction score was higher in patients with NARES than in those without NARES (AR, LAR, or IR) (*p* < 0.01). Consistent with the findings of a previous study,^6^ the LEs were positive for patients with AR, NARES, and LAR, whereas it was negative for patients with IR. The local sIgE level was positive for patients with AR or LAR, whereas it was negative for patients with IR and NARES. Serum sIgE was positive in patients with AR, whereas it was negative in the other groups (data not shown).

**Table 1:** Demographic and clinical features of chronic rhinitis patients and control subjects.

	Control	AR	NARES	LAR	IR	*p*-value
No. of subjects	18	28	17	11	19	
Age (y)	32.2 ± 6.4	33.7 ± 8.6	40.7 ± 9.6	38.6 ± 7.5	38.6 ± 9.2	0.072
Gender (M/F)	10/8	17/11	9/8	4/7	8/11	0.453
Asthma	0	7	4	1	0	0.093
Nasal obstruction, median (IQR)	–	8 (7–9)	5 (5–7)	4 (3–5)	5 (5–7)	**0.000**
Rhinorrhea, median (IQR)	–	4 (4–5)	4 (2–5)	4 (1–4)	4 (3–5)	0.112
Nasal itching, median (IQR)	–	3 (2–4)	2 (1–3)	2 (0–3)	2 (1–3)	0.084
Sneezing, median (IQR)	–	3 (2–4)	2 (1.5–3.5)	2 (1–3)	4.5 (3–5)	**0.001**
Loss of smell, median (IQR)	–	2 (1–3)	3 (2–3.5)^a^	4 (3–4)	0 (0–1)	**0.000**
VAS score, median (IQR)	–	20 (17.25–22.75)	17 (14–18)	14 (12–16)	17 (15–19)	**0.000**

AR, allergic rhinitis; F, female; IgE, immunoglobulin E; IR, idiopathic rhinitis; LAR, local allergic rhinitis; M, male; NARES, nonallergic rhinitis with eosinophilia syndrome; y, years. The boldface presents the significant difference among AR, NARES, LAR, and IR groups.

a With significant difference between patients with and without NARES (*p* < 0.01)

**Fig. 1:**
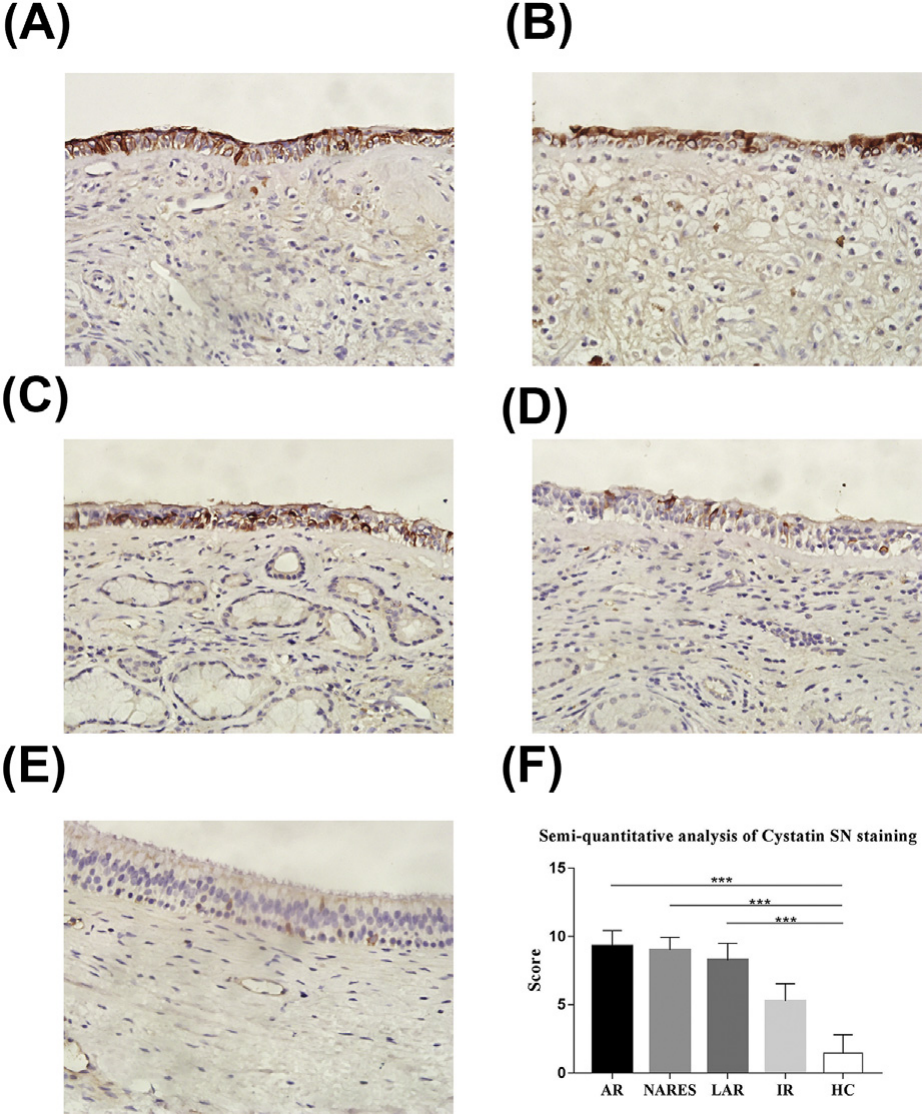
Localization of Cystatin SN between different groups. In the AR (A), NARES (B), LAR (C), IR (D), and control (E) groups (H&E, 400 × magnification). (F) Semiquantitative analysis of stained Cystatin SN in the control subjects and patients with AR, NARES, LAR, and IR (for each group, n = 5). The data are presented as mean ± standard deviation (SD). ∗∗∗: *p* < 0.001. AR, allergic rhinitis; NARES, nonallergic rhinitis with eosinophilia syndrome; LAR, local allergic rhinitis; IR, idiopathic rhinitis

**Fig. 2:**
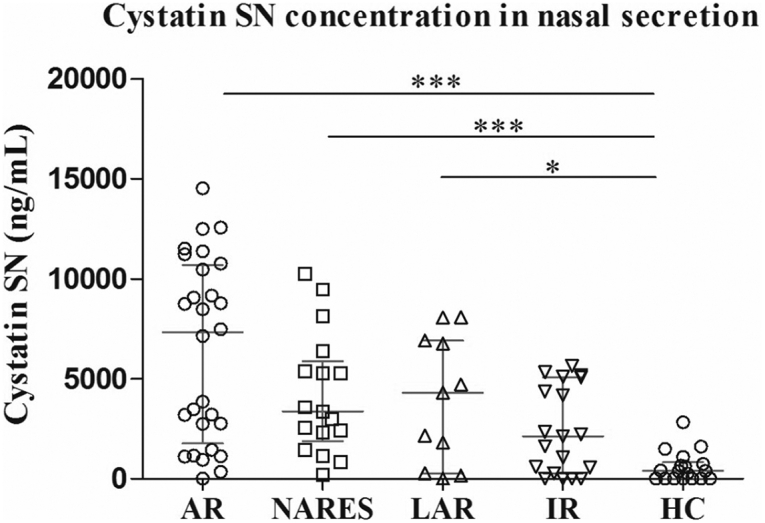
Measurements of Cystatin SN in the nasal fluid of patients with AR, NARES, LAR, and IR and control subjects. AR, allergic rhinitis; NARES, nonallergic rhinitis with eosinophilia syndrome; LAR, local allergic rhinitis; IR, idiopathic rhinitis; HC, health control. The data are presented as median ± interquartile range (IQR). ∗∗∗: *p* < 0.001. ∗: *p* < 0.05

**Fig. 3:**
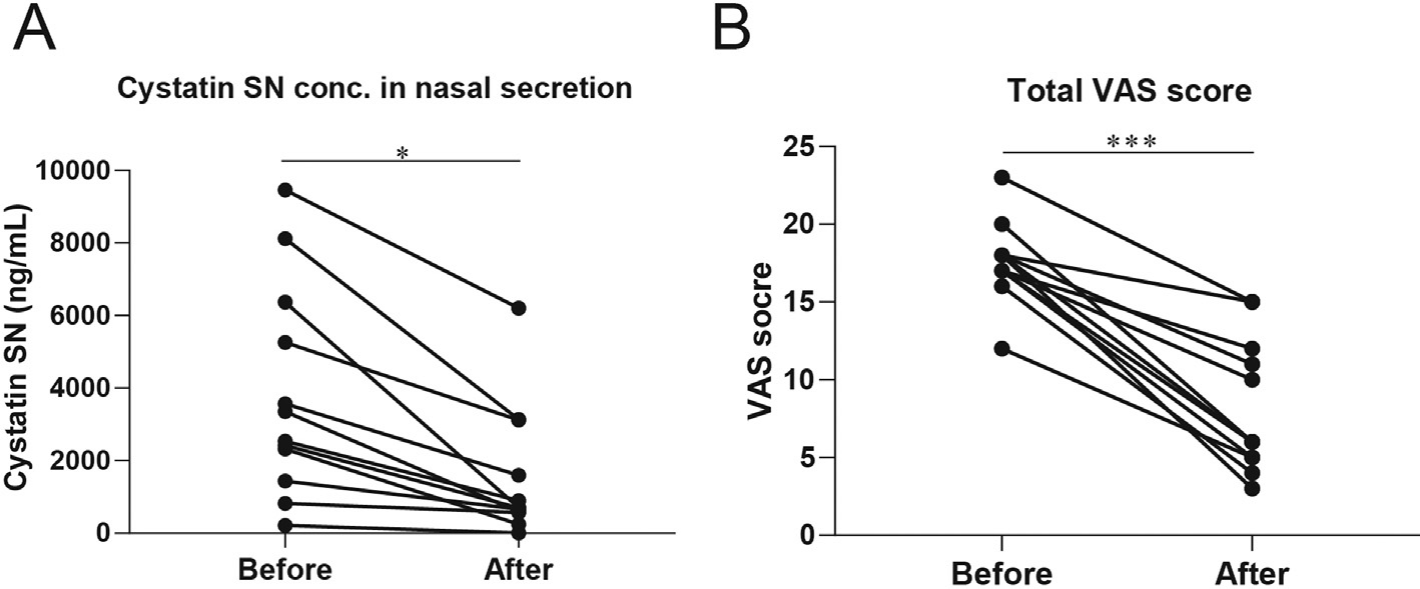
Comparison of Cystatin SN (A) and VAS score (B) before and after the medical intervention (a combination of 2 sprays, 64 μg budesonide in the morning (1 spray per nostril; total, 128 μg per day) and 1 tablet of 10 mg montelukast). VAS, visual analogue scale. ∗∗∗: *p* < 0.001. ∗: *p* < 0.05

Cystatin SN is mainly expressed on the epithelial cells of patients with AR, NARES, and LAR (Fig. 1). The Cystatin SN concentration in nasal secretion was significantly higher in patients with AR, NARES, and LAR than in the healthy controls, but they were not significantly different between patients with IR and the control subjects (Fig. 2). After 2 weeks of treatment, there was a significant decrease in the Cystatin SN level and VAS score in the NARES group compared with the baseline data (Fig. 3A and B).

### Predictive models for NARES

All metrics found to be significantly different based on between-group comparative analysis, including nasal obstruction score, sneezing score, olfaction score, LEs, local sIgE, serum sIgE, and Cystatin SN concentration, were introduced to Spearman correlation coefficient analysis to evaluate the correlations between the parameters. As shown in Table 2, several parameters presented moderate or weak correlations, whereas the correlation between local sIgE and serum sIgE was high (*r* > 0.7). To further exclude the possible collinearity, the variance inflation factor (VIF) for the parameters was detected. The VIF of serum sIgE was higher than 4. Considering that the olfaction score was significantly higher in patients with NARES than in those without NARES, two logistic regression models were established: model 1 includes nasal olfaction score and concentration of Cystatin SN together with serum sIgE; and in model 2, serum sIgE was replaced by local sIgE, and the other parameters were unchanged.

**Table 2:** Correlation coefficient and significant difference among variables determined by Spearman analysis.

	Loss of smell	Sneezing	CST1 conc.	Blood sIgE	Local sIgE	Local eosinophils
Nasal obstruction	−0.119	0.002	0.318∗∗	0.632 ∗∗∗	0.265 ∗	0.152
Loss of smell	–	−0.500 ∗∗∗	0.127	−0.048	0.292∗	0.622 ∗∗∗
Sneezing	–	–	−0.109	−0.029	−0.224	−0.458 ∗∗∗
CST1 conc.	–	–	–	0.328∗∗	0.273∗∗	0.324 ∗∗
Blood sIgE	–	–	–	–	0.742 ∗∗∗	0.450 ∗∗∗
Local sIgE	–	–	–	–	–	0.606 ∗∗∗

∗: *p* < 0.05; ∗∗: *p* < 0.01; ∗∗∗: *p* < 0.001; conc., concentration

**Fig. 4:**
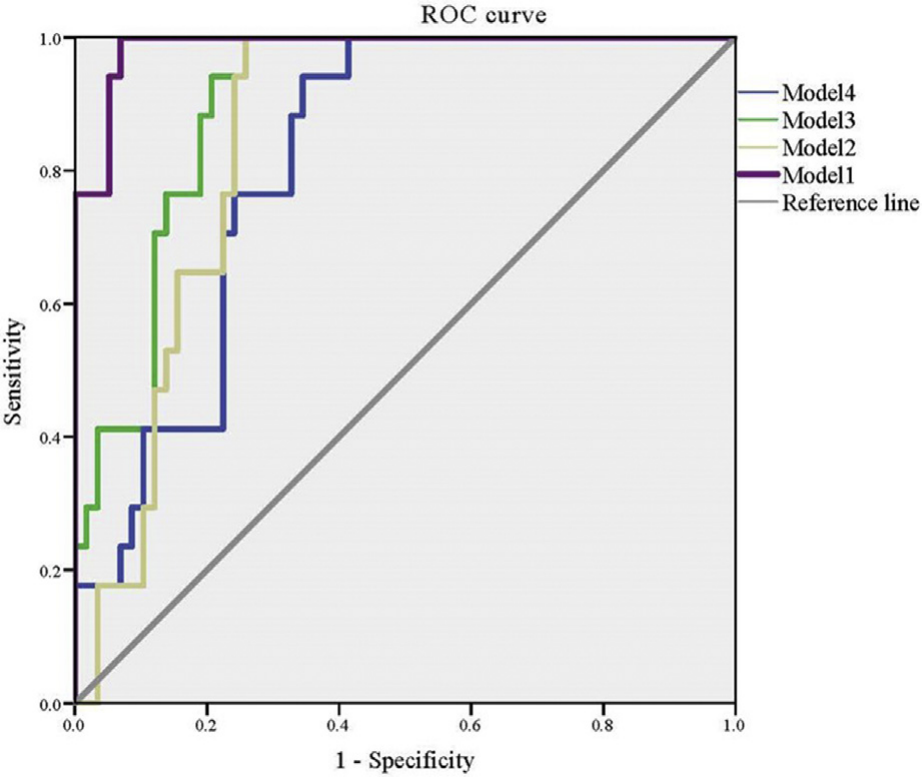
Receiver operating characteristic (ROC) curves of different models. Model 1, local sIgE combined with Cystatin SN and loss of smell. Model 2, serum sIgE combined with Cystatin SN and loss of smell. Model 3, local sIgE combined with Cystatin SN. Model 4, serum sIgE combined with Cystatin SN

As shown in Table 3, the AUC of model 1 was 0.987 and that of model 2 was 0.850. There was a significant difference between these two AUC values (*p* = 0.001; Table 4, Fig. 4), which indicated that the model of Cystatin SN and smell loss score together with local sIgE presented a high predictive accuracy for NARES than with serum sIgE.

**Table 3:** Receiver operating characteristic curve analysis of factors associated with NARES and the sensitivity and specificity of clinical markers for diagnosing NARES.

Models	*p*-value	AUC	95%CI Lower	95%CI Upper
Model 1	0.000	0.987	0.968	1.000
Model 2	0.000	0.850	0.766	0.934
Model 3	0.000	0.902	0.834	0.969
Model 4	0.000	0.815	0.720	0.911

AUC, area under the curve; CI, confidence interval.

Model 1, local sIgE combined with Cystatin SN and loss of smell.

Model 2, serum sIgE combined with Cystatin SN and loss of smell.

Model 3, local sIgE combined with Cystatin SN.

Model 4, serum sIgE combined with Cystatin SN.

**Table 4:** Significant differences (*p*-value) among each model.

	Model 2	Model 3	Model 4
Model 1	**0.001**	**0.008**	**0.000**
Model 2	–	–	0.421
Model 3			**0.009**

Model 1, local sIgE combined with Cystatin SN and loss of smell.

Model 2, serum sIgE combined with Cystatin SN and loss of smell.

Model 3, local sIgE combined with Cystatin SN.

Model 4, serum sIgE combined with Cystatin SN. The boldface presented the significant difference between the two groups.

Next, we determined whether the introduction of symptoms is necessary. The collaborative predictive values of local sIgE and Cystatin SN with or without olfaction score were evaluated. As shown in Table 3, Table 4, local sIgE and Cystatin SN combined with olfaction presented the highest AUC value, and a significant difference was observed compared with the other models. The optimal values of sensitivity and specificity were 1 and 0.931, respectively. However, if symptom is not taken into consideration, the combination of Cystatin SN with local sIgE is superior to serum sIgE in predicting NARES (Table 4). The goodness-of-fit for the models was investigated by the Hosmer-Lemeshow test, the *p*-values for all models are larger than 0.05 (Supplementary Table 1).

## Discussion

NARES is a type of classical nonallergic rhinitis whose prevalence is usually underestimated. Nonetheless, its prevalence is reported to be between 2% and 33%.^6^, ^7^, ^8^^,^^11^ NARES is highly associated with the entire airway, and eosinophils are pivotal in its pathophysiology. However, the diagnostic criteria of eosinophils vary from 5% to 25%^7^^,^^8^^,^^13^, ^14^, ^15^ and are difficult to use in daily work. Therefore, relatively stable biomarkers are required to diagnose NARES.

Cystatin SN is a member of the type 2 cystatin protein superfamily. Recent studies have proposed that members of the cystatin superfamily may be involved in a number of immunological processes^26^, ^27^, ^28^ and Cystatin SN has been reported to increase in childhood respiratory allergy and seasonal allergic rhinitis.^20^^,^^29^ Yan et al.,^30^ demonstrated that Cystatin SN is significantly increased in the tissue of patients with eosinophilic chronic rhinosinusitis with nasal polyps (ECRSwNP) and decreased in the tissues of patients without ECRSwNP. Besides, the levels of both *CST1* mRNA and protein were found to be positively correlated with the percentage of tissue eosinophils and FeNO levels in patients with ECRSwNP.^30^ These results suggest that Cystatin SN serves as a marker of eosinophilic nasal disease but its function in the pathology of NARES is yet to be fully understood. Consistent with these findings, the Cystatin SN level was significantly higher in patients with NARES than in other non-allergic rhinitis groups and controls, indicating that Cystatin SN might be a novel and useful biomarker for eosinophilic airway inflammation.

The migration and activity of eosinophils are pivotal in the pathophysiology of NARES, and the role of eicosanoids in its inflammatory process has been evaluated.^10^ Besides intranasal corticosteroid (INS), we administered 10 mg montelukast to patients with NARES for 2 weeks and found that compared with the baseline, the Cystatin SN level and VAS score were significantly decreased. Therefore, we could demonstrate that Cystatin SN is a reliable biomarker for not only NARES diagnosis but also medical treatment efficacy assessment. Moreover, our findings suggest that besides the use of INS, anti-leukotrienes, which could effectively reduce eosinophilic inflammation, should be emphasized for patients with NARES.

This study has several implications. First, models of Cystatin SN combined with local sIgE could used to diagnose NARES with an accuracy of higher than 0.9, indicating Cystatin SN could serve as an alternative biomarker of LEs to diagnose NARES. Based on this result, the development of kits using gold immunochromatographic assay (GICA) strips for the rapid detection of Cystatin SN in nasal secretions in the future may help save labor. Second, patients with NARES were found to exhibit several clinical characteristics including nasal congestion, rhinorrhea, nasal itching, and sneezing, which are similar to those observed in allergic rhinitis. In this cohort, Cystatin SN combined with local sIgE and olfaction presented a predictive efficacy similar to that of model 2, which included nasal obstruction, olfaction, sneezing, Cystatin SN, and local sIgE. Furthermore, a moderate correlation was observed between olfaction and LEs (*p* < 0.001, *r* = 0.622, Table 2). Thus, the study indicated that for CR patients assessed with a loss of smell, LEs or Cystatin SN concentration should be recommended to clarify diagnosis. However, in the presence of AR and LAR, neither IgE nor Cystatin SN could serve as an independent biomarker to diagnose NARES.

In the present study, we included 75 patients with CR and no significant differences were observed in age, sex ratio, and onset of asthma between the groups. These results are not in full agreement with those of previous studies, in which NARES was demonstrated to display female predominance.^11^^,^^31^^,^^32^ It was interesting to note that there was no significant difference between the AR and NARES groups, which were highly associated with eosinophilic airway inflammation, compared with other groups regarding the onset of asthma. This is because some earlier studies have suggested that asthma was highly associated with allergic and nonallergic rhinitis.^33^, ^34^, ^35^, ^36^, ^37^, ^38^ However, these outcomes might be due to the fewer samples collected in the present study. The LEs were significantly higher in the NARES groups than in the other non-allergic rhinitis groups, suggesting that non-IgE mediated eosinophilic inflammation may manifest in both upper and lower airways as NARES and eosinophilic asthma, respectively. This discovery aligns with the well-documented “one airway, one disease” concept associated with whole airway allergy symptoms. However, the morbidity of asthma in the present cohort was lower than that found in a previous study.^36^

The current study was limited by the sample size; thus, a multicenter study with a larger sample size is needed in the future to confirm the findings of the present study.

## Conclusions

In conclusion, the study indicated that Cystatin SN in nasal secretions was upregulated in patients with NARES compared with the controls. The combination of Cystatin SN, local sIgE, and olfaction presented an optimistic efficiency for the diagnosis of NARES.

## Availability of data and materials

The datasets generated during the current study are available from the corresponding author on reasonable request.

## Acknowledgment

None.

## Consent for publication

All authors agreed to publication of the work.

## Funding

This work was supported by grants from the National Natural Science Foundation of China (81900916), Beijing Municipal Administration of Hospitals’ Youth Programme (QML20190208), the Priming Scientific Research Foundation for the Senior Researcher in Beijing TongRen Hospital, Capital Medical University (2017-YJJ-GGL-005), 10.13039/501100012166National Key R&D Program of China (2018YFC0116800), the program for Changjiang Scholars and Innovative Research Team (IRT13082), Beijing Scientific and Technological Overall Plan (Z171100000117002), 10.13039/501100009331Beijing Municipal Administration of Hospitals Clinical Medicine Development of Special Funding Support (XMLX201816), 10.13039/501100004826Beijing Natural Science Foundation (7194247), Public Welfare Development and Reform Pilot Project (2019-10), and Beijing Municipal Administration of Hospitals incubating Program (PX20190007).

## Ethics approval

The study was approved by the Medical Ethics Committee of Beijing TongRen Hospital (version 1.0). All patients provided written informed consent before enrollment and data collection.

## Authors' contributions

All authors significantly contributed to the study. YM and BY prepared the manuscript and performed statistical analyses. BY, YW, and DW collected the data. CW and LZ designed and supervised the study.

## Declaration of Competing Interest

The authors report no competing interests.
