# Supplementary material for: Diagnosis and management of nonallergic rhinitis with eosinophilia syndrome using cystatin SN together with symptoms
Source: World Allergy Organ J. 2020 Jun 17;13(7):100134. doi: 10.1016/j.waojou.2020.100134 (PMC7301177; doi:10.1016/j.waojou.2020.100134)
Supplement: Multimedia component 1 [file mmc1.docx]

**Supplementary Table 1.** The *p*-values of the Hosmer-Lemeshow test for each model

| **Models** | ***p*-value** |
| --- | --- |
| **Model 1** | 0.997 |
| **Model 2** | 0.585 |
| **Model 3** | 0.723 |
| **Model 4** | 0.635 |
